# Development and Experimental Validation of Real Fluid Models for CFD Calculation of ORC and Steam Turbine Flows

**DOI:** 10.3390/ma14226879

**Published:** 2021-11-15

**Authors:** Andrii Rusanov, Roman Rusanov, Piotr Klonowicz, Piotr Lampart, Grzegorz Żywica, Aleksandra Borsukiewicz

**Affiliations:** 1 A.M. Pidhornyi Institute of Mechanical Engineering Problems of the National Academy of Sciences of Ukraine, 61046 Kharkiv, Ukraine; rusanov@ipmach.kharkov.ua (A.R.); roman_rusanov@ipmach.kharkov.ua (R.R.); 2 Institute of Fluid Flow Machinery, Polish Academy of Sciences, 80-231 Gdansk, Poland; lampart@imp.gda.pl (P.L.); gzywica@imp.gda.pl (G.Ż.); 3 ORC Power Plants Research and Development Centre, West Pomeranian University of Technology, 70-310 Szczecin, Poland; aborsukiewicz@zut.edu.pl

**Keywords:** 3D CFD, equation of state, turbine flow, steam turbine, ORC turbine

## Abstract

The article describes an interpolation–analytical method of reconstruction of the IAPWS-95 equations of state and the modified Benedict–Webb–Rubin equations of state with 32 terms (mBWR32). The method enables us to provide the thermodynamic closure in 3D computational fluid dynamics (CFD) calculations of turbomachinery flows with real working media, such as steam and Organic Rankine Cycle (ORC) fluids. The described approach allows for the sufficient accuracy of 3D flow calculations and does not require a significant increase in computational cost over perfect gas calculations. The method is validated against experimental data from measurements and compared with computational results from the model using the Tammann equation of state. Three turbine blading systems are considered—a multi-stage configuration from a low-pressure cylinder of a large-power steam turbine and two ORC microturbines working with organic media HFE7100 and R227ea. The calculation results obtained using the described method of approximation of the IAPWS-95 and mBWR32 equations exhibit satisfactory agreement with the experimental data, considering pressures, temperatures and enthalpies in key sections, as well as turbine power and efficiency in a wide range of changing thermodynamic parameters. In contrast, the Tammann equation of state provides acceptable results only for relatively small changes of thermodynamic parameters.

## 1. Introduction

Computational fluid dynamics (CFD) methods are widely used in various fields of science and technology: power engineering, aviation and astronautics, chemical industry, oil and gas industry, etc. A leading approach in CFD for gas dynamics and turbomachinery applications is based on numerical integration of the Reynolds-averaged Navier–Stokes (RANS) equations. In order to close RANS equations and establish a relation between the thermodynamic quantities, equations of state are used, the choice of which depends essentially on the reliability of the obtained computational results.

Currently, the most common equations of state used in 3D calculations are the perfect gas, Tammann, and Van der Waals equations [1,2,3,4]. In many cases, their use is justified and provides acceptable results. A significant increase in the accuracy of the obtained results can be achieved with an individual selection of the equations’ constants, as appropriate for the actual range of change of thermodynamic parameters.

However, when the processes occur in a wide range of thermodynamic conditions (as in multi-stage steam turbines or in ORC turbines with a large pressure ratio), or when a phase transition takes place, the simulation accuracy can be significantly reduced [4]. Then, it is necessary to apply more complex equations of state that are polynomials with a large number of terms. Examples of such equations are the IAPWS-95 equations of state [5] and various forms or modifications of the Benedict–Webb–Rubin equations [6,7,8,9]. The IAPWS-95 equations are used to describe the thermodynamic properties of water and steam, while the Benedict–Webb–Rubin equations of state are applied to a wider range of working fluids and are among basic equations of the USA National Institute of Standards and Technology [10]. Unfortunately, direct use of these equations in 3D flow calculations is currently impossible, because in this case, the computing processor time increases by 1.5–2 orders of magnitude.

In response to this question, in the present paper, an interpolation–analytical method to represent thermodynamic functions in complex state equations such as IAPWS-95 equations of state and Benedict–Webb–Rubin equations of state is proposed. The use of this method in 3D CFD calculations allows us to ensure sufficient computational accuracy and, on the other hand, does not require a significant increase in computational costs over perfect gas computations. Here, one of the most accurate modifications of the Benedict–Webb–Rubin thermal equation of state that has 32 terms is used. A method for the determination of constants of the modified Benedict–Webb–Rubin equation of state with 32 terms, on the basis of the available fields of thermodynamic values, is described. These constants of the mBWR32 equation are given for fluids HFE7100 [11] and R227ea [12] working in the investigated ORC microturbines.

Three-dimensional (3D) CFD calculations of the flow parts of a low-pressure cylinder (LPC) of a large power steam turbine and two ORC microturbines are carried out with the help of the software package IPMFlow [13]. The results of CFD calculations obtained with the proposed reconstruction of the IAPWS–95 and mBWR32 equations are compared with the available experimental data from the considered turbines and also with the computational results obtained using the Tammann equation of state. The main purpose of these studies is the validation of the proposed approach of approximating complex thermodynamic equations of state rather than a detailed analysis of the thermodynamic processes and flow phenomena occurring in the investigated turbomachines. In addition, admissible intervals of change of thermodynamic parameters, for which the use of Tammann equation of state can yield reasonable results, are established.

## 2. Mathematical Model

### 2.1. Flow Solver

Three-dimensional (3D) CFD calculations are performed using the IPMFlow software package [13], which is the evolution of earlier software packages FlowER and FlowER–U [14]. It implements the following elements in the mathematical model: Reynolds-averaged unsteady Navier–Stokes equations [15,16], Menter’s *k*-*ω* SST two-equation turbulence model [17], the finite volume method, and the implicit quasi-monotone high-order ENO-scheme [18]. Such an approach to provide 3D CFD is typical for calculations of turbine flow parts [19,20]. In most cases for steady flows, the computational domain includes one blade-to-blade channel of each blade row, with periodic boundary conditions. At the boundaries between adjacent blade rows, values of thermodynamic parameters are averaged in the circumferential direction and exchanged. To speed up the convergence process, a quasi-multigrid algorithm [21] and an individual time step are used. Usually, a block-structured H-type grid is used for calculations. The domain of tip leakage is meshed, whereas the domain of shroud leakage is not meshed, and the so-called sink-source approach is used. This approach enables extractions and injections of the fluid streams from and into the main flow domain, so it can be applied to over-the-shroud leakages, inter-disk leakages, and technological extractions [22]. In order to obtain computational results with acceptable accuracy, it was found sufficient to use about 500,000 cells (elementary volumes) in one blade-to-blade channel in each row and a mesh resolution near walls that provided y^+^ values below 2. Every time, final computations were proceeded by grid independence checks.

### 2.2. Equations of State

The equation of perfect gas and the caloric equations of state obtained on its basis [23,24] are most widely known and most often used in 3D CFD calculations. Using the perfect gas equation, it is possible to carry out CFD calculations in flow parts with a relatively short expansion/compression line, such as in a single stage of a high or medium pressure steam turbine cylinder in the superheated steam zone, in one stage of an air compressor of a gas turbine engine, or in a single-stage industrial fan, etc. The constants of the equation of state in such calculations are usually determined by the “average” thermodynamic values between the inlet and outlet.

The Tammann thermal equation of state is quite simple but at the same time more accurate:

(1) $$P+p_{0}=\rho RT.$$

It differs from the perfect gas equation by the presence of an additional constant. The caloric equations of state obtained on the basis of the thermal equation of state are described in detail in the paper [25]. The extensive experience of the authors has shown that for the calculation of turbomachinery flows, the constants for the Tammann equation should be determined from values of total temperature, pressure and density at the inlet to the flow domain, as well as isentropic static values of temperature, pressure and density at the outlet of the computational domain. Then, the constants R, $p_{0}$, and γ can be determined from Equation (1) and the following relation:

$$\frac{P+p_{0}}{\rho^{\gamma }}=const.$$

The IAPWS–95 equation is nowadays the most accurate equation of state that describes the properties of water and steam. Its detailed description and the corresponding caloric equations of state are presented in [5].

The modified Benedict–Webb–Rubin thermal equation of state with 32 terms has the following form [26,27,28]:

(2) $$P=\rho RT+\rho^{2}\left[G(1)T+G(2)T^{1/2}+G(3)+\frac{G(4)}{T}+\frac{G(5)}{T^{2}}\right]+\rho^{3}\left[G(6)T+G(7)+\frac{G(8)}{T}+\frac{G(9)}{T^{2}}\right]\;+\rho^{4}\left[G\left(10\right)T+G\left(11\right)+\frac{G\left(12\right)}{T}\right]+\rho^{5}\left[G\left(13\right)\right]+\rho^{6}\left[\frac{G\left(14\right)}{T}+\frac{G\left(15\right)}{T^{2}}\right]+\rho^{7}\left[\frac{G\left(16\right)}{T}\right]\;+\rho^{8}\left[\frac{G\left(17\right)}{T}+\frac{G\left(18\right)}{T^{2}}\right]+\rho^{9}\left[\frac{G\left(19\right)}{T^{2}}\right]+\rho^{3}\left[\frac{G\left(20\right)}{T^{2}}+\frac{G\left(21\right)}{T^{3}}\right]\exp\left(\gamma \rho^{2}\right)\;+\rho^{5}\left[\frac{G\left(22\right)}{T^{2}}+\frac{G\left(23\right)}{T^{4}}\right]\exp\left(\gamma \rho^{2}\right)+\rho^{7}\left[\frac{G\left(24\right)}{T^{2}}+\frac{G\left(25\right)}{T^{3}}\right]\exp\left(\gamma \rho^{2}\right)+\rho^{9}\left[\frac{G\left(26\right)}{T^{2}}+\frac{G\left(27\right)}{T^{4}}\right]\exp\left(\gamma \rho^{2}\right)\;+\rho^{11}\left[\frac{G(28)}{T^{2}}+\frac{G(29)}{T^{3}}\right]\exp\left(\gamma \rho^{2}\right)+\rho^{13}\left[\frac{G(30)}{T^{2}}+\frac{G(31)}{T^{3}}+\frac{G(32)}{T^{4}}\right]\exp\left(\gamma \rho^{2}\right).$$

The above equation and its corresponding forms have a very complex shape. The caloric equation and equations describing thermodynamic functions corresponding to the BWR32 equation are enclosed and described in detail in Appendix A.

### 2.3. Determination of Constants of the Benedict–Webb–Rubin Equation of State

Usually, constants for equations of state, including mBWR32, are determined based on the experimental data. In the literature, one can find information about constants of simple equations of state for various types of working fluids. Open information about values of constants for the mBWR32 equation of state is available only for a few working fluids. However, there are various software packages, for example REFPROP [10] that allows us to calculate the array of fields of thermodynamic functions *y_i_* for a fairly wide range of working fluids. Based on them, it is possible to determine the constants for the working fluids of our interest.

The gas constant *R* is determined as a ratio of the universal gas constant to the molecular weight of the considered working fluid. The remaining constants *γ* and *G(i)* are determined using the least squares method to assure the smallest square deviation of the dimensionless unknown function from the array base point values:

(3) $$\sum_{i=1}^{n}{\left(\frac{f_{i}-y_{i}}{y_{i}}\right)}^{2}\to min,$$

where *f_i_*—the required thermodynamic function of the mBWR32 equation of state at point *i* of the array; *y_i_*—value of the thermodynamic function at point *i*, obtained using REFPROP software package; *n*—dimension of the base points array. Problem (2) can be solved in the following way: if *γ* is assumed as known and constant, then the condition (2) can be replaced by the condition:

(4) $$\sum_{i=1}^{n}\left(\frac{f_{i}-y_{i}}{y_{i}^{2}}\frac{\partial f_{i}}{\partial G_{j}}\right)=0,\;j=1..37.$$

Expression (3) forms a system of 37 linear equations with respect to 37 unknowns *G (j)* for the thermodynamic functions: pressure, Helmholtz free energy, entropy and the partial derivative of pressure to density at constant temperature. The linear system of Equation (3) is solved by the Gauss method with dominant diagonal terms. The accuracy of calculations is set at a quadruple precision with 32 characters. Such a large mantissa is needed to maintain the required high accuracy. The global search for the solution of the problem defined by Equation (2) is carried out by varying *γ* in the range:

$$-K\rho_{*}^{2}\leq \gamma \leq K\rho_{*}^{2}\;\mathrm{and}\;K=100\;\frac{m^{6}}{kg^{2}}$$

where ρ_*_ is the value of density at the critical point. Constants are found for the simultaneous fulfilment of condition (2) for the following thermodynamic functions: pressure, the Helmholtz free energy, entropy, and the partial derivative of pressure to density at constant temperature.

The values of the constants of the mBWR32 equation, obtained using the method described above, for water vapour, HFE7100, and R227ea as working fluids in the investigated turbines are summarized in Appendix B. As initial data, thermodynamic arrays obtained using the REFPROP [10] program were taken at (1) 1100 points in the entire range of variation and 73 points on the saturation line for temperature from 280 to 800 K and pressure up to 96 MPa for water vapour, (2) 610 points in the entire range of variation and 35 points on the saturation line for temperature from 300 to 600 K and pressure up to 3 MPa for HFE-7100, and (3) 800 points in the entire range of variation and 45 points on the saturation line for temperature from 150 to 470 K and pressure up to 8.6 MPa for R227ea working fluid.

### 2.4. Method of Interpolation–Analytical Representation of Thermodynamic Functions

As mentioned earlier, the direct use of complex equations of state (IAPWS-95, mBWR32, etc.) in numerical algorithms for 3D viscous flow calculations leads to an increase in computational time by 1.5–2 orders of magnitude, which is unacceptable. To reduce computational cost, an interpolation–analytical method of representation of thermodynamic functions is used. This method was first applied to take into account the thermodynamic properties of water and steam in 3D calculations based on the IAPWS-95 equation of state in [29]. According to this approach, the required thermodynamic functions are determined from the following dependencies:

$$T=\frac{p}{\rho RZ_{t}};\;\rho =\frac{p}{h}Z_{\rho };\;u=\frac{p}{\rho }\frac{Z_{u}}{Z_{t}};\;p=\rho \cdot u\cdot Z_{p};\;a=\sqrt{\frac{p}{\rho }\frac{Z_{a}}{Z_{t}}};\;h=\frac{p}{\rho }\left(1+\frac{Z_{u}}{Z_{t}}\right);\;C_{V}=R\cdot Z_{Cv};\;S=R\cdot Z_{S};\;u_{p}=\frac{Z_{u}}{\rho \cdot Z_{t}};\;\;u_{\rho }=-\frac{p}{\rho }\frac{Z_{u}}{Z_{t}};\;S_{p}=\frac{C_{V}}{p};\;T_{p}=\frac{1}{\rho R\cdot Z_{t}};\;T_{\rho }=-\frac{p}{\rho^{2}R\cdot Z_{t}},$$

where *Z_t_ = Z_t_(ρ,p), Z_ρ_ = Z_ρ_(h,p), Z_u_ = Z_u_(ρ,p), Z_p_ = Z_p_(ρ,u), Z_Cv_ = Z_Cv_(ρ,p), Z_Cp_ = Z_Cp_(ρ,p),* and *Z_S_ = Z_S_(ρ,p)* are the dimensionless compressibility coefficients for the corresponding thermodynamic functions. These are determined by interpolation from pre-calculated arrays of the base points. This approach has significant advantages over the “direct” interpolation of thermodynamic functions, which is used in some software packages [30,31]. This is due to the fact that the range of variation of the dimensionless compressibility coefficients is much narrower, and unlike the required thermodynamic functions, the dimensionless compressibility coefficients are monotonic functions of the assumed dependent variables. Due to this, arrays of base points of much smaller dimensions can be stored to ensure the acceptable interpolation accuracy. Furthermore, to reduce the dimensions of the arrays without sacrificing the accuracy, the independent variables of pressure and density are considered in a logarithmic scale. To interpolate the compressibility coefficients, polynomials of the third degree are used. Values of the dimensionless compressibility coefficients are defined in the base points as:

$$Z_{t}=\frac{p}{\rho RT},\;Z_{u}=\frac{u}{RT},\;Z_{\rho }=\frac{h\rho }{p},\;Z_{p}=\frac{p}{\rho \cdot u},\;Z_{a}=\frac{a^{2}}{RT},\;Z_{Cv}=\frac{C_{v}}{R},\;Z_{Cp}=\frac{C_{p}}{R},\;Z_{S}=\frac{S}{R},$$

where the corresponding values of *p, ρ, T, u, h, a, c_p_, c_v_* and *S* are calculated using the thermal equations of state (IAPWS-95, mBWR32, etc.). A more detailed description of the interpolation method has been presented in the work of Rusanov et al. [29].

## 3. Comparison of Computational Results and Experimental Data

### 3.1. Low-Pressure Cylinder of 360 MW Steam Turbine

**Figure 1:**
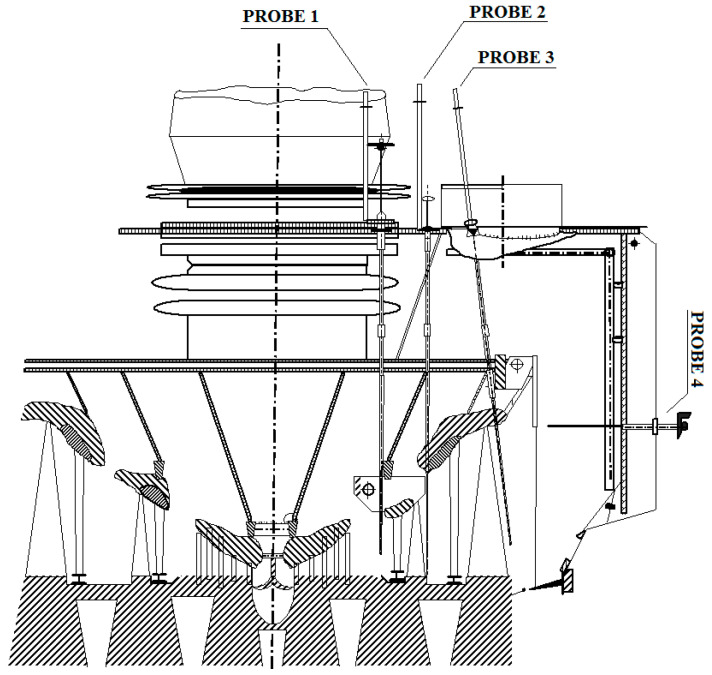
View of the flow part in LPC of a 360 MW steam turbine.

The steam flow in the low-pressure cylinder (LPC) of a 360 MW turbine was investigated experimentally and numerically. A view of the investigated LPC is shown in Figure 1. Thermodynamic parameters were measured in span-wise distribution in axial diffusers behind stages 3, 4, and 5 by probes 1, 2, and 3, respectively and in the exit diffuser by probe 4, as shown in Figure 1. Details of the experimental study and measurement method were described in earlier publications [4,32].

A numerical study of this LPC using various equations of state was first presented in [4]. In that paper, CFD results obtained using the equation of state for a perfect gas with constant and variable specific heats were compared and validated against experimental data. It was shown that specifying the heat capacities as linear functions of temperature brought the obtained results closer to the experiment; however, this approach significantly worsened the stability of the computational code. The results of calculations of this LPC using the IAPWS-95 equation of state and their comparison with computational results based on the perfect gas equation and Van der Waals equation of state were shown in [29]. A fundamental improvement in the coincidence of experimental data and computational results while using the IAPWS-95 equation of state was illustrated.

In this study, the LPC is investigated numerically with the help of CFD code IPMFlow, making use of three equations of state—the Tammann equation, the IAPWS-95 equation, and the mBWR32 equation. To carry out the calculations, the following boundary conditions are assumed—the total pressure and temperature at the inlet, and the static pressure at the outlet (see Table 1). The calculations take into account two recuperative extractions after stages 3 and 4, with the mass extraction of 6 and 5.5 kg/s respectively, as well as inter-disk and over-the-shroud leakages [4]. The fifth stage rotor blade is unshrouded, so there is a meshed radial gap available for tip leakage flow.

The constants of the Tammann equation of state are determined from values of total thermodynamic parameters at the inlet and isentropic ones at the outlet (Table 1). The values of constants of the Tammann equation and the isentropic enthalpy difference between the inlet and outlet of the flow part obtained on its basis are presented in Table 2. The isentropic enthalpy drop obtained from the Tammann equation of state is 9.2% greater than that obtained from the IAPWS-95 and mBWR32 equations of state.

Table 3 shows a comparison of the calculation results with experimental data, including pressures, temperatures, enthalpies, and flow rates at the outlet of the subsequent stages 3, 4, and 5. It can be seen that the differences between the experimental data and calculation results obtained using the IAPWS95 and mBWR32 equations of state are insignificant. The greatest difference is observed for the static temperature behind the third stage, −1.2%; in the other cases, the differences are much smaller than 1%, which is in favour of the presented computational method. The differences between the experimental data and calculation results obtained with the help of the Tammann equation of state are much more significant and, in some cases, reach 15%. However, it should be noted that the results obtained using the Tammann equation are more accurate compared to the perfect gas equation with constant heat capacities and similar in quality to the results obtained using the perfect gas equation with variable specific heats [4,29].

**Figure 2:**
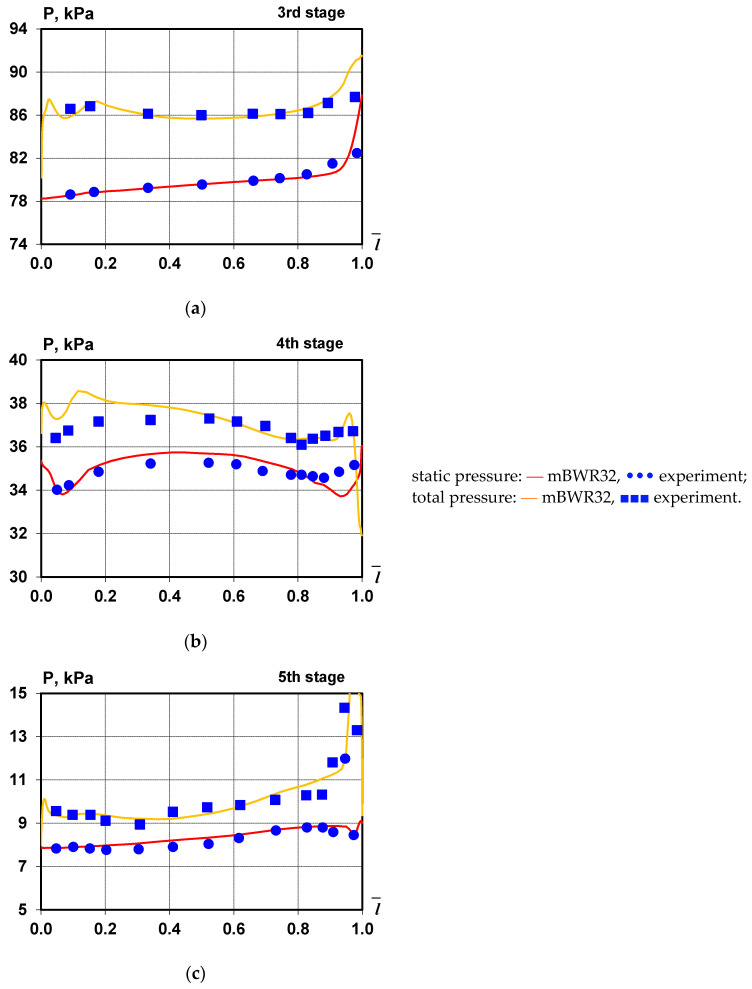
Total and static pressure distributions along the blade beyond the third (**a**), fourth (**b**), and fifth (**c**) stages.

**Figure 3:**
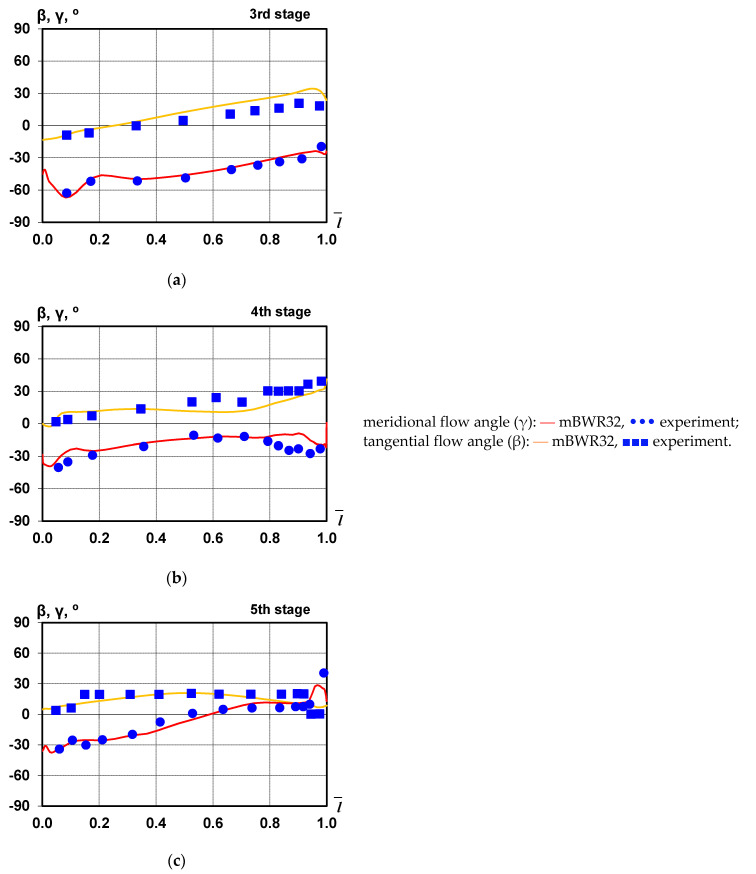
Meridional (γ) and tangential (β) flow angles distributions along the blade beyond the third (**a**), fourth (**b**), and fifth (**c**) stages.

Figure 2 and Figure 3 show a comparison of distributions of static and total pressure, meridional, and tangential flow angles downstream of stages 3, 4, and 5 of the investigated large-power steam turbine, which were obtained experimentally using the mBWR32 equations of state. The computed values are averaged along the circumference. A good agreement between experimental and calculation results can be observed both in the main flow area and in the regions of influence of tip/shroud leakage.

### 3.2. Radial ORC Turbine with HFE7100 Working Fluid

**Figure 4:**
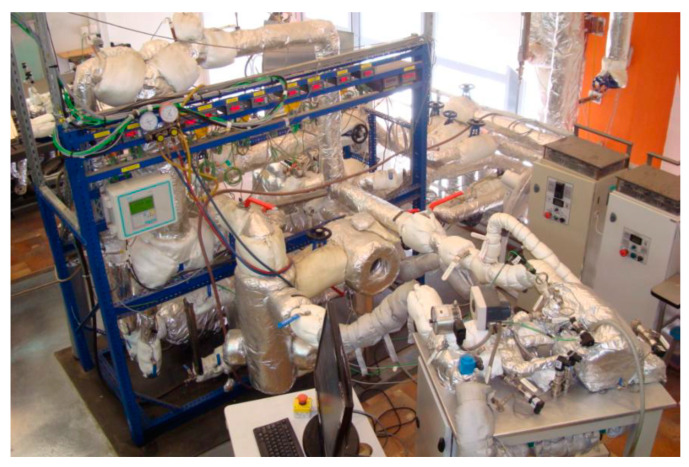
View of the experimental ORC power plant at IMP PAN.

A 2.5 kW ORC micro power plant working on organic fluid HFE7100 supplied from the wood chips boiler was developed at the Institute of Fluid Flow Machinery, Polish Academy of Sciences (IMP PAN) as a model cogeneration unit dedicated for household applications. A general view of the micro-power plant is shown in Figure 4. A detailed description of the plant with its operational experience and the results of experimental studies can be found in the papers [33,34,35].

**Figure 5:**
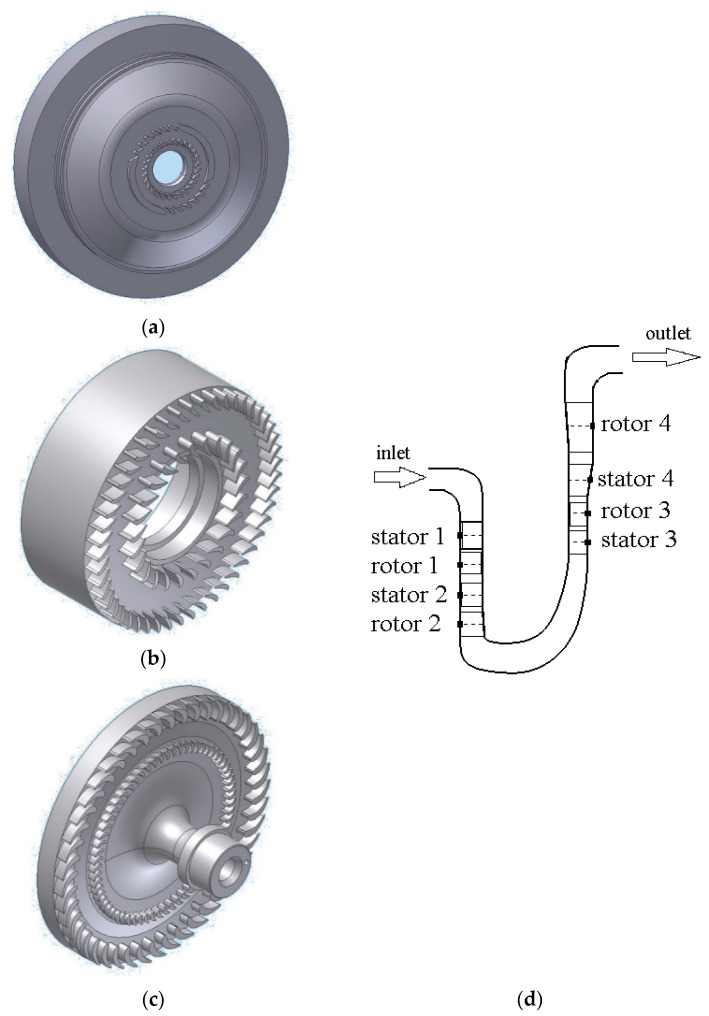
CAD model of a 2.5 kW ORC turbine: (**a**) stator of stage 1 and 2, (**b**) rotor of stage 1 and 2, (**c**) rotor of stage 3 and 4, (**d**) meridional section of the microturbine flow part.

The main element of this cogeneration unit is a multi-stage radial microturbine. The turbine consists of four stages, where the first two stages are centripetal (radial inward), and the other two are centrifugal (radial outward). The turbine has small dimensions; the outlet edges of the fourth-stage rotor blades are located at a diameter of 74.5 mm (relative to the turbine rotation axis). The stator and rotor blades from all stages have tip gaps near the meridional contours of size 0.15 mm. The first stage is designed with partial admission supply (degree of partial admission − 0.5). The design rotational velocity is equal to 20,000 rpm. A view of the individual elements and meridional section of the ORC microturbine flow part is illustrated in Figure 5.

Numerical studies of microturbine flow and comparison of computational results with the experimental data are carried out for three operational regimes. Boundary conditions for these regimes, including inlet total temperatures and pressures, as well as outlet static pressures, rotational speeds, and isentropic parameters are provided in Table 4. The calculations were made using two equations of state—the Tammann and mBWR32 equation. The Tammann equation constants are determined from total parameters at the inlet and isentropic parameters at the outlet as in Table 4. Values of the Tammann equation constants and the isentropic enthalpy difference between the inlet and outlet of the flow part obtained on its basis are presented in Table 5. It can be seen that the maximum difference between the isentropic enthalpy drop obtained from the Tammann equation of state is 8% compared to that found from the REFPROP [10] software.

The comparisons of calculated results and experimental data are shown in Table 6. In the experiment, the power and, accordingly, the efficiency were measured at the generator terminals. The power and efficiency of the microturbine calculated in CFD studies were corrected taking into account the generator efficiency whose average value was determined at 85%. As it can be seen from the presented comparison, the results obtained using the mBWR32 equation of state are much closer to the experimental data than those obtained using the Tammann equation. For the mBWR32 equation, the maximum discrepancy between the experiment and computation in mass flow is 6.5%, in power, it is –2.7% and in efficiency, it is –4.1%. At the same time, for the Tammann equation of state, those discrepancies are 10.9%, 7.4%, and 6.2%, respectively.

### 3.3. Axial ORC Turbine with R227ea Working Fluid

**Figure 6:**
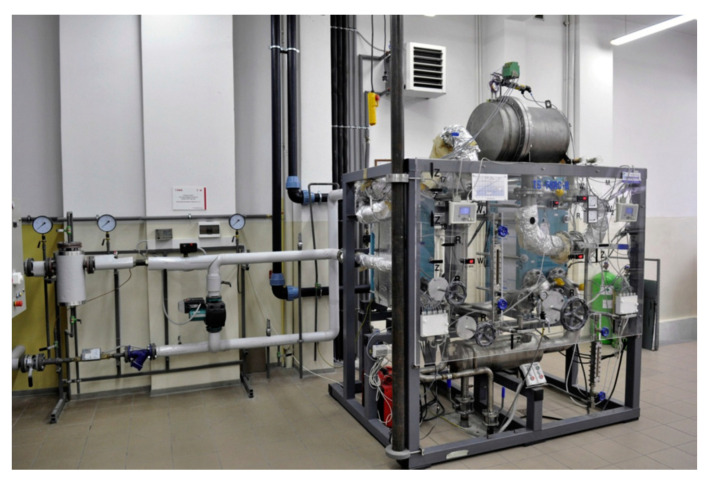
Picture of the ORC power plant.

An ORC plant working with R227ea was developed at the West Pomeranian University of Technology in Szczecin as a model installation for geothermal applications. This ORC unit is supplied by hot water from the district heating network. A general view of the ORC power plant is presented in Figure 6, whereas its more detailed description is given in the papers [35,36].

**Figure 7:**
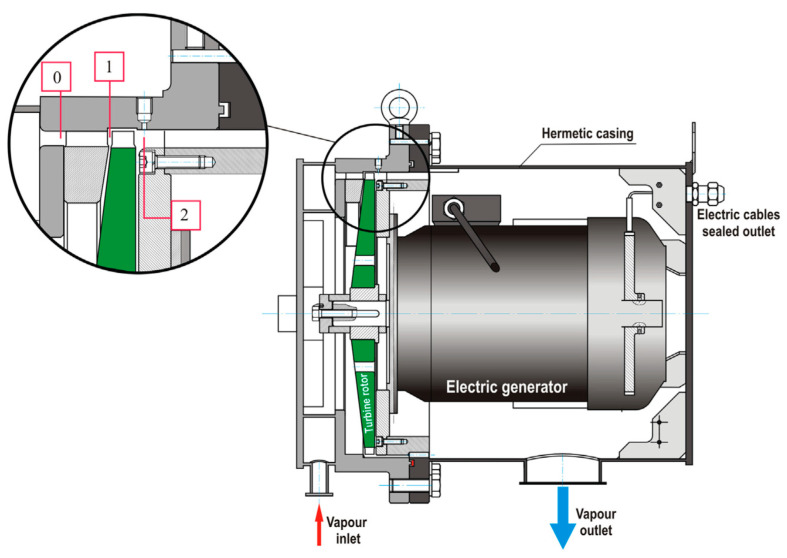
View of the ORC turbogenerator.

The ORC cycle is equipped with an axial single-stage microturbine with a degree of partial admission equal to 1/9. Turbine blades have low heights: 9.7 mm—stator blade and 11.4 mm—rotor blade. A view on the ORC turbogenerator and turbine flow path is shown in Figure 7. The small size of the turbine and a high degree of partial admission make its CFD calculation a non-trivial task.

CFD investigations of the microturbine flow and comparison of computational results with the experimental data were carried out for four operational modes. Boundary conditions, including inlet total temperatures and pressures as well as outlet static pressures, rotational speeds, and isentropic parameters are provided in Table 7. Calculations were conducted for two equations of state—the Tammann and mBWR32 equations. The Tammann equation constants determined from the total parameters at the inlet and isentropic parameters at the outlet, together with the isentropic enthalpy difference between the inlet and outlet of the flow part, are presented in Table 8. The maximum difference for the isentropic enthalpy drop obtained from the Tammann equation of state is 3% compared to that found from the REFPROP [10] software.

Table 9 shows the comparison of calculation results and experimental data. In the experiment, the power was measured at the generator terminals. Thus, the power and efficiency of the microturbine calculated in CFD studies were corrected, taking into account the generator efficiency whose average value was determined this time at 91%.

From the presented results, it can be seen that, in contrast to the previous examples, there is no explicit advantage of using the mBWR32 equation of state. Generally, there is a satisfactory agreement between the experimental data and calculation results obtained from both equations of state. According to the authors, this is due to a relatively small drop in thermodynamic parameters in the considered flow part, which leads to an insignificant difference in isentropic enthalpy drops obtained during the calculations using the mBWR32 and Tammann equations of state (less than 3%). It can be concluded that for small isentropic enthalpy drops, the Tammann equation of state satisfactorily describes the thermodynamic properties of the working fluid.

## 4. Conclusions

The method of interpolation–analytical reconstruction of the complex IAPWS-95 and mBWR32 equations of state was presented to take into account the real properties of working fluids in 3D CFD calculations. The method for determination of the constants of the modified Benedict–Webb–Rubin equation of state with 32 terms was proposed. The constants of the mBWR32 equation were calculated for the HFE-7100 and R227ea working fluids.

The results of the 3D flow calculation in the LPC of a steam turbine and ORC microturbines with the HFE7100 and R227ea working fluids were presented. Calculations were performed using the Tammann equation and the proposed interpolation–analytical method of representation of the IAPWS-95 and mBWR32 equations of state. The comparison of the calculation results with experimental data showed satisfactory agreement in the case of the IAPWS-95 and mBWR32 equations for a wide range of change of thermodynamic parameters. It was found that the Tammann equation of state provides acceptable results for relatively small drops in thermodynamic parameters in the flow part, when the isentropic enthalpy drop obtained during the calculations using the Tammann equation differs from the exact value by not more than 3–4%.

The method is able to improve the quality of flow modelling in thermal turbomachinery, especially in terms of the flow of fluids, the behaviour of which differs significantly from that of an ideal gas. This functionality was not available in earlier versions of the in-house IPMFlow software.

## Figures and Tables

**Table 1:** Inlet/outlet boundary conditions and isentropic quantities.

$P_{in}^{*},\;\mathrm{kPa}$	$T_{in}^{*},\;K$	$\rho_{in}^{*},\;\mathrm{kg}/m^{3}$	*P_ex_*, kPa	$T_{isex},\;K$	$\rho_{isex},\;\mathrm{kg}/m^{3}$	$\Delta H_{is},\;\mathrm{kJ}/\mathrm{kg}$
519	539	2.12	8.3	315.21	0.06485	699.2

**Table 2:** Constants of the Tammann equation of state and the resultant isentropic enthalpy drop.

γ	*R*, J/(kg·K)	$p_{0},\;\mathrm{kPa}$	$\Delta H_{isT}$, kJ/kg
1.154	455.1	1.002	763.85

**Table 3:** Comparison of experimental and calculated pressures, temperatures, enthalpies, and flow rates at the outlets from stages 3, 4, and 5.

Parameter	Experiment	Tammann	IAPWS95	mBWR32
Inlet				
P *, kPa	519			
T *, K	539			
H *, kJ/kg	2993.64			
Outlet of stage 3				
*P*, kPa	79.9	78.85	79.92	79.91
Δ*P*, %		1.3	−0.03	0.15
*T*, K	371.2	429.4	366.71	366.71
Δ*T*, %		−15.7	1.21	1.21
*H*, kJ/kg	2647	2647.2	2643.51	2643.51
Δ*h*, %		−0.01	0.13	0.13
*G*, kg/s	107.9	103.8	107.63	107.64
Δ*G*, %		3.8	0.25	0.24
Outlet of stage 4				
*P*, kPa	34.9	34.87	35.04	35.02
Δ*P*, %		0.09	−0.4	−0.34
*T*, K	346.4	390.58	345.88	345.87
Δ*T*, %		−12.75	0.15	0.15
*H*, kJ/kg	2531	2503.5	2525.14	2527.14
Δ*h*, %		1.09	0.23	0.23
*G*, kg/s	100.9	97.6	101.11	101.12
Δ*G*, %		3.27	−0.21	−0.22
Outlet of stage 5				
*P*, kPa	8.3			
Δ*P*, %		0.0	0.0	0.0
*T*, K	314.8	334.63	315.38	315.38
Δ*T*, %		−6.3	−0.19	−0.19
*H*, kJ/kg	2350	2326.9	2353.27	2353.27
Δ*h*, %		0.98	−0.14	−0.14
*G*, kg/s	96	91.39	95.81	95.81
Δ*G*, %		4.81	0.2	0.2

**Table 4:** ORC microturbine operational regimes. Inlet/outlet boundary conditions and isentropic quantities.

№	Ω, rpm	$P_{in}^{*},\;\mathrm{kPa}$	$T_{in}^{*},\;K$	$\rho_{in}^{*},\;{\mathrm{kg}/m}^{3}$	$P_{ex},\;\mathrm{kPa}$	$T_{isex},\;K$	$\rho_{isex},\;{\mathrm{kg}/m}^{3}$	$\Delta H_{is},\;\mathrm{kJ}/\mathrm{kg}$
1	18,120	760.2	427	75.07	203.2	403.32	16.56	15.16
2	19,620	786.8	416.1	85.73	192.7	390.06	16.34	15.28
3	19,000	750	413.7	80.84	175	387.41	14.83	188

**Table 5:** Constants of the Tammann equation of state and the resultant isentropic enthalpy drop.

№	γ	*R*, J/(kg·K)	$p_{0},\;\mathrm{kPa}$	$\Delta H_{isT},\;\mathrm{kJ}/\mathrm{kg}$	$\frac{\left|\Delta H_{isT}-\Delta H_{is}\right|}{\Delta H_{is}}100$%
1	1.038	21.96	−56.6	14.29	5.75
2	1.038	20.31	−63.35	14.09	7.76
3	1.039	20.77	−55.76	14.62	7.93

**Table 6:** Comparison of experimental and calculated microturbine flow rate, power, and efficiency for given operational regimes as defined in Table 4.

Operational Mode	Experiment/Computation	*G,* kg/s	*N,* kW	*η,* %
1	Experiment	0.1796	1.8188	66.82
	Tammann	0.1955 (+8.86%)	1.9539 (+7.43%)	69.96 (+3.14%)
	mBWR32	0.1882 (+4.81%)	1.8604 (+2.29%)	65.21 (−1.61%)
2	Experiment	0.189	2.0824	72.05
	Tammann	0.2098 (+10.88%)	1.9461 (−6.54%)	65.84 (−6.21%)
	mBWR32	0.2014 (+6.45%)	2.0889 (+0.32%)	67.98 (−4.07%)
3	Experiment	0.185	1.9353	65.88
	Tammann	0.2031 (+9.8%)	2.0257 (+4.67%)	68.21 (+2.34%)
	mBWR32	0.1913 (+3.41%)	1.9877 (+2.71%)	65.51 (−0.36%)

**Table 7:** ORC microturbine operational regimes. Inlet/outlet boundary conditions and isentropic quantities.

№	Ω, rpm	$P_{in}^{*},\;\mathrm{kPa}$	$T_{in}^{*},\;K$	$\rho_{in}^{*},\;\mathrm{kg}/m^{3}$	$P_{ex},$ kPa	$T_{isex},\;K$	$\rho_{isex},\;\mathrm{kg}/m^{3}$	$\Delta H_{is},\;\mathrm{kJ}/\mathrm{kg}$
1	2847	820.8	322.4	64.96	320.8	299.29	24.13	12.28
2	2895	831.8	321.4	66.58	315.8	297.45	23.92	12.56
3	3128	916.8	326	73.42	342.8	301.18	25.75	12.82
4	3264	953.8	326.8	77.07	332.8	300.1	25.04	13.68

**Table 8:** Constants of the Tammann equation of state and the resultant isentropic enthalpy drop.

№	γ	*R*, J/(kg·K)	$P_{0},\;\mathrm{kPa}$	$\Delta H_{isT},\;\mathrm{kJ}/\mathrm{kg}$	$\frac{\left|\Delta H_{isT}-\Delta H_{is}\right|}{\Delta H_{is}}100\%$
1	1.075	36.465	−57.617	12.05	1.83
2	1.076	36.151	−58.74	12.3	2.05
3	1.076	35.497	−67.68	12.53	2.25
4	1.076	35.166	−68.7	13.32	2.67

**Table 9:** Comparison of experimental and calculated microturbine flow rate, power and efficiency for given operational regimes, as defined in Table 7.

Operation Mode	Experiment/Computation	*G,* kg/s	*N,* kW	*η,* %
1	Experiment	1.111	8.451	61.94
	Tammann	1.166 (+4.99%)	8.41 (−0.49%)	55.821 (−6.12%)
	mBWR32	1.142 (+2.79%)	8.264 (−2.21%)	55.059 (−6.88%)
2	Experiment	1.154	8.67	59.819
	Tammann	1.19 (+3.14%)	8.809 (+1.60%)	56.216 (−3.60%)
	mBWR32	1.161 (+0.62%)	8.637 (−0.38%)	55.432 (−4.39%)
3	Experiment	1.311	10.022	59.63
	Tammann	1.309 (−0.16%)	10.210 (+1.87%)	57.61 (−2.02%)
	mBWR32	1.275 (−2.77%)	10.034 (+0.12%)	56.912 (−2.72%)
4	Experiment	1.334	10.846	59.434
	Tammann	1.373 (+2.95%)	11.506 (+6.08%)	58.009 (−1.43%)
	mBWR32	1.330 (−0.28%)	11.348 (+4.63%)	57.637 (−1.80%)

## Data Availability

Data is contained within the article.

## Nomenclature

*a* [m/s]	sonic speed
*C_p_* [J/(kg×K)]	isobaric heat capacity
*C_v_* [J/(kg×K)]	isochoric heat capacity
*f* [J/kg]	Helmholtz free energy
*G* [kg/s]	mass flow rate
*G(i)* […] ^†^	constants of the mBWR32 equation, ^†^ constants of the mBWR32 equation are dimensional; their dimensions depend on particular terms at which these constants appear
*H* [J/kg]	enthalpy
*N* [kW]	power
*P* [kPa]	pressure
*P** [kPa]	total pressure
*p_o_* [kPa]	constant of the Tammann equation of state
*R* [J/(kg×K)]	gas constant
*S* [J/(kg×K)]	entropy
*T* [K]	temperature
*T** [K]	total temperature
*u* [J/kg]	internal energy
*γ* [–]	adiabatic index
*η* [%]	efficiency
*ρ* [kg/m^3^]	density
*ρ** [kg/m^3^]	density in critical point
Ω [rpm]	rotational speed

## Indexes

*in*	parameters at the inlet
*ex*	parameters at the outlet
*is*	isentropic parameters
*isT*	isentropic parameters obtained using the Tammann equation of state
*	stagnant flow parameters

## Appendix A. Determination of the Caloric Equation and Equations Describing Thermodynamic Functions Corresponding to the Modified Benedict–Webb–Rubin Thermal Equation of State

To determine the caloric equations of state, differential equations of thermodynamics [29,30], Equation (2), and also the dependence of the Helmholtz free energy *f* are used. The connection between the Helmholtz free energy and Equation (2) is established by the expression:

(A1) $$P=\rho^{2}{\left(\frac{\partial f}{\partial \rho }\right)}_{T}.$$

Equation (A1) allows us to express the Helmholz free energy *f* in the form of an arbitrary polynomial with respect to *T*. In this paper, the expression for the Helmholtz free energy is taken in the form:

(A2) $$f=f_{i}+f_{v},$$

where

$$f_{i}=RTln\left(\rho \right)+\rho \left[G\left(1\right)T+G\left(2\right)\sqrt{T}+G\left(3\right)+\frac{G\left(4\right)}{T}+\frac{G\left(5\right)}{T^{2}}\right]+\frac{\rho^{2}}{2}\left[G\left(6\right)T+G\left(7\right)+\frac{G\left(8\right)}{T}+\frac{G\left(9\right)}{T^{2}}\right]\;+\frac{\rho^{3}}{3}\left[G\left(10\right)T+G\left(11\right)+\frac{G\left(12\right)}{T}\right]+\frac{\rho^{4}}{4}G\left(13\right)+\frac{\rho^{5}}{5}\left[\frac{G\left(14\right)}{T}+\frac{G\left(15\right)}{T^{2}}\right]+\frac{\rho^{6}}{6}\frac{G\left(16\right)}{T}\;+\frac{\rho^{7}}{7}\left[\frac{G\left(17\right)}{T}+\frac{G\left(18\right)}{T^{2}}\right]+\frac{\rho^{8}}{8}\frac{G\left(19\right)}{T^{2}}+\frac{G(33)}{T}+G(34)T+G(35)T^{2}+G(36)Tln(T)+G(37);$$

$$f_{V}=I_{1}\left[\frac{G\left(20\right)}{T^{2}}+\frac{G\left(21\right)}{T^{3}}\right]+I_{2}\left[\frac{G\left(22\right)}{T^{2}}+\frac{G\left(23\right)}{T^{4}}\right]+I_{3}\left[\frac{G\left(24\right)}{T^{2}}+\frac{G\left(25\right)}{T^{3}}\right]+I_{4}\left[\frac{G\left(26\right)}{T^{2}}+\frac{G\left(27\right)}{T^{4}}\right]\;+I_{5}\left[\frac{G\left(28\right)}{T^{2}}+\frac{G\left(29\right)}{T^{3}}\right]+I_{6}\left[\frac{G\left(30\right)}{T^{2}}+\frac{G\left(31\right)}{T^{3}}+\frac{G\left(32\right)}{T^{4}}\right];\;I_{1}=\int \rho \exp\left(\gamma \rho^{2}\right)d\rho =\frac{1}{2\gamma }\exp\left(\gamma \rho^{2}\right);\;I_{2}=\int \rho^{3}\exp\left(\gamma \rho^{2}\right)d\rho =\frac{\gamma \rho^{2}-1}{2\gamma^{2}}\exp(\gamma \rho^{2});\;I_{3}=\int^{\;}\rho^{5}\exp\left(\gamma \rho^{2}\right)d\rho =\frac{\gamma^{2}\rho^{4}-2\gamma \rho^{2}+2}{2\gamma^{3}}\exp(\gamma \rho^{2});\;I_{4}=\int \rho^{7}\exp\left(\gamma \rho^{2}\right)d\rho =\frac{\gamma^{3}\rho^{6}-3\gamma^{2}\rho^{4}+6\gamma \rho^{2}-6}{2\gamma^{4}}\exp(\gamma \rho^{2});\;I_{5}=\int \rho^{9}\exp\left(\gamma \rho^{2}\right)d\rho =\frac{\gamma^{4}\rho^{8}-4\gamma^{3}\rho^{6}+12\gamma^{2}\rho^{4}-24\gamma \rho^{2}+24}{2\gamma^{5}}\exp(\gamma \rho^{2});\;I_{6}=\int \rho^{11}\exp\left(\gamma \rho^{2}\right)d\rho =\frac{\gamma^{5}\rho^{10}-5\gamma^{4}\rho^{8}+20\gamma^{3}\rho^{6}-60\gamma^{2}\rho^{4}+120\gamma \rho^{2}-120}{2\gamma^{6}}\exp(\gamma \rho^{2}).$$

The function *f_i_* is a component of the Helmholtz free energy containing non-exponential terms, whereas *f_v_* contains exponential terms. *I_1_* -*I_6_* are density integrals of the exponential terms. An additional polynomial consisting of five terms is introduced into Equation (A2), which leads to an increase in the number of constants *G* from 32 to 37. The introduction of additional components increases the accuracy in determination of the Helmholtz free energy and other thermodynamic functions.

In view of the form of Equations (2) and (A2), the required thermodynamic functions take the form given below:

Internal energy:

$$u=f-T{\left(\frac{\partial f}{\partial T}\right)}_{\rho },$$

where ${\left(\frac{\partial f}{\partial T}\right)}_{\rho }={\left(\frac{\partial f_{i}}{\partial T}\right)}_{\rho }+{\left(\frac{\partial f_{v}}{\partial T}\right)}_{\rho }$;

$${\left(\frac{\partial f_{i}}{\partial T}\right)}_{\rho }=Rln\left(\rho \right)+\rho \left[G\left(1\right)+\frac{1}{2}\frac{G\left(2\right)}{\sqrt{T}}-\frac{G\left(4\right)}{T^{2}}-2\frac{G\left(5\right)}{T^{3}}\right]+\frac{\rho^{2}}{2}\left[G\left(6\right)-\frac{G\left(8\right)}{T^{2}}-2\frac{G\left(9\right)}{T^{3}}\right]\;+\frac{\rho^{3}}{3}\left[G\left(10\right)-\frac{G\left(12\right)}{T^{2}}\right]+\frac{\rho^{5}}{5}\left[-\frac{G\left(14\right)}{T^{2}}-2\frac{G\left(15\right)}{T^{3}}\right]-\frac{\rho^{6}}{6}\frac{G\left(16\right)}{T^{2}}+\frac{\rho^{7}}{7}\left[-\frac{G\left(17\right)}{T^{2}}-2\frac{G\left(18\right)}{T^{3}}\right]\;-\frac{\rho^{8}}{4}\frac{G\left(19\right)}{T^{3}}-\frac{G(33)}{T^{2}}+G(34)+2G(35)T+G(36)+G(36)ln(T);$$

$${\left(\frac{\partial f_{v}}{\partial T}\right)}_{\rho }=I_{1}\left[-2\frac{G\left(20\right)}{T^{3}}-3\frac{G\left(21\right)}{T^{4}}\right]+I_{2}\left[-2\frac{G\left(22\right)}{T^{3}}-4\frac{G\left(23\right)}{T^{5}}\right]+I_{3}\left[-2\frac{G\left(24\right)}{T^{3}}-3\frac{G\left(25\right)}{T^{4}}\right]\;+I_{4}\left[-2\frac{G\left(26\right)}{T^{3}}-4\frac{G\left(27\right)}{T^{5}}\right]+I_{5}\left[-2\frac{G\left(28\right)}{T^{3}}-3\frac{G\left(29\right)}{T^{4}}\right]+I_{6}\left[-2\frac{G\left(30\right)}{T^{3}}-3\frac{G\left(31\right)}{T^{4}}-4\frac{G\left(32\right)}{T^{5}}\right]$$

.

Isochoric heat capacity:

$$c_{V}={\left(\frac{\partial u}{\partial T}\right)}_{\rho }={\left(\frac{\partial f}{\partial T}\right)}_{\rho }-{\left(\frac{\partial f}{\partial T}\right)}_{\rho }-T{\left(\frac{\partial^{2}f}{\partial T^{2}}\right)}_{\rho }=-T{\left(\frac{\partial^{2}f}{\partial T^{2}}\right)}_{\rho },$$

where ${\left(\frac{\partial^{2}f}{\partial T^{2}}\right)}_{\rho }={\left(\frac{\partial^{2}f_{i}}{\partial T^{2}}\right)}_{\rho }+{\left(\frac{\partial^{2}f_{V}}{\partial T^{2}}\right)}_{\rho }$;

$${\left(\frac{\partial^{2}f_{i}}{\partial T^{2}}\right)}_{\rho }=\rho \left[-\frac{1}{4}\frac{G\left(2\right)}{T^{\frac{3}{2}}}+2\frac{G\left(4\right)}{T^{3}}+6\frac{G\left(5\right)}{T^{4}}\right]+\frac{\rho^{2}}{2}\left[2\frac{G\left(8\right)}{T^{3}}+6\frac{G\left(9\right)}{T^{4}}\right]+\frac{2}{3}\rho^{3}\frac{G\left(12\right)}{T^{3}}\;+\frac{\rho^{5}}{5}\left[2\frac{G\left(14\right)}{T^{3}}+6\frac{G\left(15\right)}{T^{4}}\right]+\frac{\rho^{6}}{3}\frac{G\left(16\right)}{T^{3}}+\frac{\rho^{7}}{7}\left[2\frac{G\left(17\right)}{T^{3}}+6\frac{G\left(18\right)}{T^{4}}\right]+\frac{3}{4}\rho^{8}\frac{G\left(19\right)}{T^{4}}\;+2\frac{G(33)}{T^{3}}+2G(35)+\frac{G(36)}{T};\;{\left(\frac{\partial^{2}f_{V}}{\partial T^{2}}\right)}_{\rho }=I_{1}\left[6\frac{G\left(20\right)}{T^{4}}+12\frac{G\left(21\right)}{T^{5}}\right]+I_{2}\left[6\frac{G\left(22\right)}{T^{4}}+20\frac{G\left(23\right)}{T^{6}}\right]+I_{3}\left[6\frac{G\left(24\right)}{T^{4}}+12\frac{G\left(25\right)}{T^{5}}\right]\;+I_{4}\left[6\frac{G\left(26\right)}{T^{4}}+20\frac{G\left(27\right)}{T^{6}}\right]+I_{5}\left[6\frac{G\left(28\right)}{T^{4}}+12\frac{G\left(29\right)}{T^{5}}\right]+I_{6}\left[6\frac{G\left(30\right)}{T^{4}}+12\frac{G\left(31\right)}{T^{5}}+20\frac{G\left(32\right)}{T^{6}}\right].$$

Isobaric heat capacity:

$$c_{P}={\left(\frac{\partial h}{\partial T}\right)}_{P}={\left[\frac{\partial }{\partial T}\left(u+\frac{P}{\rho }\right)\right]}_{P}={\left(\frac{\partial u}{\partial T}\right)}_{P}-\frac{P}{\rho^{2}}{\left(\frac{\partial \rho }{\partial T}\right)}_{P}={\left(\frac{\partial f}{\partial T}\right)}_{P}-{\left(\frac{\partial f}{\partial T}\right)}_{\rho }-T{\left[\frac{\partial {\left(\frac{\partial f}{\partial T}\right)}_{\rho }}{\partial T}\right]}_{P}-\frac{P}{\rho^{2}}{\left(\frac{\partial \rho }{\partial T}\right)}_{P}\;=-T{\left[\frac{\partial {\left(\frac{\partial f}{\partial T}\right)}_{\rho }}{\partial T}\right]}_{P}=-T\left[{\left(\frac{\partial^{2}f}{\partial T^{2}}\right)}_{\rho }+\frac{A}{\rho^{2}}{\left(\frac{\partial \rho }{\partial T}\right)}_{P}\right]=-T\left[{\left(\frac{\partial^{2}f}{\partial T^{2}}\right)}_{\rho }-\frac{A^{2}}{\rho^{2}B}\right]=c_{V}+\frac{A^{2}T}{\rho^{2}B},$$

where

$$A=\rho R+\rho^{2}\left[G\left(1\right)+\frac{1}{2}\frac{G\left(2\right)}{\sqrt{T}}-\frac{G\left(4\right)}{T^{2}}-2\frac{G\left(5\right)}{T^{3}}\right]+\rho^{3}\left[G\left(6\right)-\frac{G\left(8\right)}{T^{2}}-2\frac{G\left(9\right)}{T^{3}}\right]\;+\rho^{4}\left[G\left(10\right)-\frac{G\left(12\right)}{T^{2}}\right]+\rho^{6}\left[-\frac{G\left(14\right)}{T^{2}}-2\frac{G\left(15\right)}{T^{3}}\right]-\rho^{7}\left[\frac{G\left(16\right)}{T^{2}}\right]+\rho^{8}\left[-\frac{G\left(17\right)}{T^{2}}-2\frac{G\left(18\right)}{T^{3}}\right]\;-2\rho^{9}\left[\frac{G\left(19\right)}{T^{3}}\right]+\rho^{3}\left[-2\frac{G\left(20\right)}{T^{3}}-3\frac{G\left(21\right)}{T^{4}}\right]\exp\left(\gamma \rho^{2}\right)+\rho^{5}\left[-2\frac{G\left(22\right)}{T^{3}}-4\frac{G\left(23\right)}{T^{5}}\right]\exp\left(\gamma \rho^{2}\right)\;+\rho^{7}\left[-2\frac{G\left(24\right)}{T^{3}}-3\frac{G\left(25\right)}{T^{4}}\right]\exp\left(\gamma \rho^{2}\right)+\rho^{9}\left[-2\frac{G\left(26\right)}{T^{3}}-4\frac{G\left(27\right)}{T^{5}}\right]\exp\left(\gamma \rho^{2}\right)\;+\rho^{11}\left[-2\frac{G\left(28\right)}{T^{3}}-3\frac{G\left(29\right)}{T^{4}}\right]\exp\left(\gamma \rho^{2}\right)+\rho^{13}\left[-2\frac{G\left(30\right)}{T^{3}}-3\frac{G\left(31\right)}{T^{4}}-4\frac{G\left(32\right)}{T^{5}}\right]\exp\left(\gamma \rho^{2}\right);\;B=RT+2\rho \left[G\left(1\right)T+G\left(2\right)\sqrt{T}+G\left(3\right)+\frac{G\left(4\right)}{T}+\frac{G\left(5\right)}{T^{2}}\right]+3\rho^{2}\left[G\left(6\right)T+G\left(7\right)+\frac{G\left(8\right)}{T}+\frac{G\left(9\right)}{T^{2}}\right]\;+4\rho^{3}\left[G\left(10\right)T+G\left(11\right)+\frac{G\left(12\right)}{T}\right]+5\rho^{4}\left[G\left(13\right)\right]+6\rho^{5}\left[\frac{G\left(14\right)}{T}+\frac{G\left(15\right)}{T^{2}}\right]+7\rho^{6}\left[\frac{G\left(16\right)}{T}\right]\;+8\rho^{7}\left[\frac{G\left(17\right)}{T}+\frac{G\left(18\right)}{T^{2}}\right]+9\rho^{8}\left[\frac{G\left(19\right)}{T}\right]+\left(3\rho^{2}+2\gamma \rho^{4}\right)\left[\frac{G\left(20\right)}{T^{2}}+\frac{G\left(21\right)}{T^{3}}\right]\exp\left(\gamma \rho^{2}\right)\;+\left(5\rho^{4}+2\gamma \rho^{6}\right)\left[\frac{G\left(22\right)}{T^{2}}+\frac{G\left(23\right)}{T^{4}}\right]\exp\left(\gamma \rho^{2}\right)+\left(7\rho^{6}+2\gamma \rho^{8}\right)\left[\frac{G\left(24\right)}{T^{2}}+\frac{G\left(25\right)}{T^{3}}\right]\exp\left(\gamma \rho^{2}\right)\;+\left(9\rho^{8}+2\gamma \rho^{10}\right)\left[\frac{G\left(26\right)}{T^{2}}+\frac{G\left(27\right)}{T^{4}}\right]\exp\left(\gamma \rho^{2}\right)+\left(11\rho^{10}+2\gamma \rho^{12}\right)\left[\frac{G\left(28\right)}{T^{2}}+\frac{G\left(29\right)}{T^{3}}\right]\exp\left(\gamma \rho^{2}\right)\;+(13\rho^{12}+2\gamma \rho^{14})\left[\frac{G(30)}{T^{2}}+\frac{G(31)}{T^{3}}+\frac{G(32)}{T^{4}}\right]\exp\left(\gamma \rho^{2}\right).$$

Entropy:

$$S=-{\left(\frac{\partial f}{\partial T}\right)}_{\rho }.$$

Sonic speed:

$$a^{2}={\left(\frac{\partial P}{\partial \rho }\right)}_{S}=\frac{T{\left(\frac{\partial P}{\partial T}\right)}_{\rho }+c_{V}{\left(\frac{\partial T}{\partial \vartheta }\right)}_{P}}{\rho^{2}c_{V}{\left(\frac{\partial T}{\partial P}\right)}_{\rho }}=\frac{T{\left(\frac{\partial P}{\partial T}\right)}_{\rho }+c_{V}\rho^{2}{\left(\frac{\partial T}{\partial \rho }\right)}_{P}}{\rho^{2}c_{V}{\left(\frac{\partial T}{\partial P}\right)}_{\rho }}=B+\frac{TA^{2}}{c_{V}\rho^{2}}.$$

## Appendix B. The Values of the Constants of the mBWR32 Equation

Table A1 shows values of the constants of the mBWR32 equation, which were obtained using the method described in Section 2.2 and Section 2.3 and the equations in Appendix A.

**Table A1:** The values of the constants of the mBWR32 equation.

Header	Water	HFE7100	R227ea
γ	−0.3231043142361733D-05	0.1205572957283814D-04	−0.4070048644587564D-05
R	461.5221	33.2578	48.900286
G(1)	0.1340188952700849D+03	−0.2525969245607927D+01	−0.3822484082371177D+00
G(2)	−0.113303814253851D+05	0.1763741711279182D+03	0.3160389924533887D+02
G(3)	0.2749007471054583D+06	−0.3601187752097783D+04	−0.6964326417230550D+03
G(4)	−0.5829440628781757D+08	0.5458851793941257D+06	0.1031623062428149D+06
G(5)	0.6411659750384605D+10	−0.5663324176871473D+08	−0.1223225314792705D+08
G(6)	0.4129629566529071D-01	0.9648702918708597D-02	−0.3193811769158057D-02
G(7)	−0.3145701008839712D+02	−0.18125682062194D+02	0.5188230632727036D+01
G(8)	0.5901184750968312D+04	0.1286291165389281D+05	−0.3213400289440099D+04
G(9)	−0.5661166428856716D+08	−0.6299375060616176D+06	0.1012250688519747D+07
G(10)	0.5132663309662063D-04	−0.8753130222925309D-05	−0.2197301359332797D-06
G(11)	−0.2658843752788538D+00	0.9307351571136587D-02	0.7172225363205859D-03
G(12)	0.1651883341198171D+03	−0.2450136776131232D+01	−0.3008513561879612D+00
G(13)	0.2266027017849203D-03	0.2632266072695997D-05	−0.1192023216555466D-06
G(14)	−0.2864338390658241D-03	0.1286131777344554D-03	0.1273328787250847D-05
G(15)	0.3051102426759072D+00	−0.4003450054558224D-01	−0.1030080706600882D-02
G(16)	−0.1031902385352661D-07	−0.7272480072620692D-06	−0.3742988522718769D-08
G(17)	0.1272432927055814D-09	0.1370553160729363D-08	0.198611771894904D-11
G(18)	−0.4205928256302565D-06	−0.7311642108133796D-07	0.8602107806419969D-09
G(19)	0.1969386059083182D-09	−0.21916813548160857D-08	−0.3439884131761577D-12
G(20)	0.5251565542126347D+08	−0.339994425757511D+07	−0.1063496697985402D+06
G(21)	0.1592357820321519D+10	0.481540423072116D+09	−0.9608289845410669D+08
G(22)	0.3910795651035839D+02	0.29141961883346D+02	−0.9382279023090581D+00
G(23)	−0.242578208092937D+07	−0.6038000612169966D+06	−0.5130819818146024D+05
G(24)	−0.2657686496886626D-03	0.9152256834775216D-04	0.2887503456985266D-05
G(25)	0.1103996250508223D-01	−0.1772968331616998D-01	−0.1133636629805082D-02
G(26)	−0.1413468432232942D-09	−0.1009669532982529D-08	−0.1517330593686373D-11
G(27)	0.1393914328000583D-04	0.5427714466691425D-04	0.2006200837684165D-06
G(28)	0.4705295923820445D-16	0.1498672827301075D-14	0.39494924918485634D-16
G(29)	−0.26690260796323977D-12	0.1907285109535534D-12	−0.1035803258385956D-13
G(30)	−0.4396223589757114D-22	−0.364152113015046D-20	−0.3003171762530103D-22
G(31)	0.1675173765276002D-18	0.1700716094002334D-17	0.1053509965613469D-19
G(32)	−0.5393086721503225D-17	−0.3340812356980171D-15	−0.4695091535355264D-19
G(33)	−0.1144301681378893D+11	−0.3397310131672742D+09	0.1148400077140449D+09
G(34)	−0.238993513584905D+06	0.3117370419539324D+04	0.9631857575641608D+04
G(35)	−0.1121389642058093D+02	−0.3284940764348829D+00	0.1552792260893027D+00
G(36)	0.310205161600632D+05	−0.5386718572092581D+03	−0.1609342678142145D+04
G(37)	0.4000508032246298D+08	0.4375477883494194D+06	−0.5838965010940802D+06

## References

[1] Younglove B.A., Ely J.F. (1987). Thermophysical Properties of Fluids II Methane, Ethane, Propane, Isobutane, and Normal Butane. J. Phys. Chem. Ref. Data.

[2] Chmielniak T.J., Wróblewski W., Dykas S. Steam flow calculations in turbine channels. Proceedings of the 3rd European Conference on Turbomachinery.

[3] Dykas S., Wróblewski W. (2012). Numerical modelling of steam condensing flow in low and high–pressure nozzles. Int. J. Heat Mass Transfer..

[4] Lampart P., Rusanov A., Yershov S., Marcinkowski S., Gardzilewicz A. (2005). Validation of 3D RANS Solver With a State Equation of Thermally Perfect and Calorically Imperfect Gas on a Multi-Stage Low-Pressure Steam Turbine Flow. Trans. ASME J. Fluids Eng..

[5] IAPWS Revised Release on the IAPWS Formulation 1995 for the Thermodynamic Properties of Ordinary Water Substance for General and Scientific Use. http://www.iapws.org.

[6] Benedict M., Webb G.B., Rubin L.C. (1940). An Empirical Equation for Thermodynamic Properties of Light Hydrocarbons and Their Mixtures: I. Methane, Ethane, Propane, and n–Butane. J. Chem. Phys..

[7] Soave G.S. (1999). An effective modification of the Benedict–Webb–Rubin equation of state. Fluid Phase Equilibria.

[8] Outcalt S.L., McLinden M.O. (1996). A modified Benedict–Webb–Rubin equation of state for the thermodynamic properties of R152a (1,1–difluoroethane). J. Phys. Chem. Ref. Data.

[9] Asano Y., Fuchizaki K. (2014). Modified Benedict–Webb–Rubin Equation of State for the Modified Lennard–Jones Fluid. J. Phys. Soc. Jpn..

[10] REFPROP National Institute of Standards and Technology, Standard Reference Database. https://www.nist.gov/srd/refprop.

[11] Rausch M.H., Kretschmer L., Will S., Leipertz A., Fröba A.P. (2015). Density, Surface, Tension, and Kinematic Viscosity of Hydrofluoroethers HFE-7000, HFE-7100, HFE7200, HFE-7300, and HFE-7500. J. Chem. Eng. Data.

[12] Lemmon E.W., Span R. (2015). Thermodynamic Properties of R-227ea, R-365mfc, R-115, and R-13I1. J. Chem. Eng. Data.

[13] Rusanov A., Rusanov R., Lampart P. (2015). Designing and updating the flow part of axial and radial-axial turbines through mathematical modelling. Open Eng..

[14] Yershov S., Rusanov A. (1996). The Application Package FlowER for the Calculation of 3D Viscous Flows through Multi-Stage Turbomachinery.

[15] Anderson D.A., Tannehill J.C., Pletcher R.H. (1986). Computational Fluid Mechanics and Heat Transfer.

[16] Fletcher C.A.J. (1988). Computational Techniques for Fluid Dynamics. Vol. I: Fundamental and General Techniques. Vol. II: Specific Techniques for Different Flow Categories.

[17] Menter F.R. (1994). Two-Equation Eddy-Viscosity Turbulence Models for Engineering Applications. AIAA J..

[18] Yershov S., Rusanov A., Gardzilewicz A., Lampart P. Calculations of 3D viscous compressible turbomachinery flows. Proceedings of the 2nd Symposium on Computational Technologies for Fluid/Thermal/Chemical Systems with Industrial Applications, ASME PVP Division Conference.

[19] Sardjono J.A., Darmawan S., Tanujaya H. (2020). Flow investigation of cross-flow turbine using CFD method. IOP Conference Series: Materials Science and Engineering.

[20] Hills N.J. Whole Turbine CFD Modelling. Proceedings of the ASME Turbo Expo 2007: Power for Land, Sea, and Air Volume 6: Turbo Expo 2007, Parts A and B.

[21] Chima R.V. (1987). Explicit multigrid algorithm for quasi-three-dimensional viscous flows in turbomachinery. J. Propuls. Power.

[22] Lampart P., Gardzilewicz A., Yershov S., Rusanov A. (2001). Investigation of interaction of the main flow with root and tip leakage flows in an axial turbine stage by means of a source/sink approach for a 3D Navier-Stokes Solver. J. Therm. Sci..

[23] Parsonage M.G. (1966). The Gaseous State.

[24] Mansour E., Desouky S., Batanoni M., Mahmoud M., Farag A., El-Dars F. (2012). Modification proposed for SRK equation of state. Oil Gas J..

[25] Rusanov A.V., Yershov S.V. (2008). Mathematical Modelling of Unsteady Gasdynamic Processes in Turbomachinery Flow Parts.

[26] Weber L.A. (1978). A Modified Benedict-Webb-Rubin Equation of State for Gaseous and Liquid Oxygen.

[27] Bridgman P.W. (1914). A complete collection of thermodynamic formulas. Phys. Rev..

[28] Lewis G.N., Randall M., Pitzer K.S., Brewer L. (1961). Thermodynamics.

[29] Rusanov A.V., Lampart P., Pashchenko N.V., Rusanov R.A. (2016). Modelling 3D steam turbine flow using thermodynamic properties of steam IAPWS–95. Pol. Marit. Res..

[30] Kunick M., Kretzschmar H.J., di Mare F., Gampe U. CFD Analysis of steam turbines with the IAPWS standard on the Spline-Based Table Look-Up Method (SBTL) for the fast calculation of real fluid properties. Proceedings of the ASME Turbo Expo, Paper GT2015-43984.

[31] Sobachkin A., Dumnov G. Numerical Basis of CAD-Embedded CFD. Proceedings of the NAFEMS World Congress 2013.

[32] Marcinkowski S. (1998). Results of Extended Flow Measurements in the LP Part of 18K370 Steam Turbine in the Belchatów Power Station.

[33] Kaczmarczyk T.Z., Zywica G., Ihnatowicz E. (2016). The experimental investigation of the biomass–fired ORC system with a radial microturbine. Appl. Mech. Mater..

[34] Kaczmarczyk T.Z., Zywica G., Ihnatowicz E. (2017). The impact of changes in the geometry of a radial microturbine stage on the efficiency of the micro CHP plant based on ORC. Energy.

[35] Klonowicz P., Borsukiewicz-Gozdur A., Hanausek P., Kryllowicz W., Brüggemann D. (2014). Design and performance measurements of an organic vapour turbine. Appl. Therm. Eng..

[36] Borsukiewicz-Gozdur A. (2013). Experimental investigation of R227ea applied as working fluid in the ORC power plant with hermetic turbogenerator. Appl. Therm. Eng..

